# Relevance of gene mutations and methylation to the growth of pancreatic intraductal papillary mucinous neoplasms based on pyrosequencing

**DOI:** 10.1038/s41598-021-04335-z

**Published:** 2022-01-10

**Authors:** Go Asano, Katsuyuki Miyabe, Hiroyuki Kato, Michihiro Yoshida, Takeshi Sawada, Yasuyuki Okamoto, Hidenori Sahashi, Naoki Atsuta, Kenta Kachi, Akihisa Kato, Naruomi Jinno, Makoto Natsume, Yasuki Hori, Itaru Naitoh, Kazuki Hayashi, Yoichi Matsuo, Satoru Takahashi, Hiromu Suzuki, Hiromi Kataoka

**Affiliations:** 1 Department of Gastroenterology and Metabolism, Nagoya City University Graduate School of Medical Sciences, 1, Kawasumi, Mizuho-Cho, Mizuho-Ku, Nagoya, 467-8601 Japan; 2 Department of Gastroenterology, Japanese Red Cross Aichi Medical Center Nagoya Daini Hospital, Nagoya, 466-8650 Japan; 3 Department of Experimental Pathology and Tumor Biology, Nagoya City University Graduate School of Medical Sciences, Nagoya, 467-8601 Japan; 4 Department of Gastroenterological Surgery, Nagoya City University Graduate School of Medical Sciences, Nagoya, 467-8601 Japan; 5 Department of Molecular Biology, Sapporo Medical University School of Medicine, Sapporo, 060-8556 Japan

**Keywords:** Cysts, Gastrointestinal cancer, Oncogenes, Tumour biomarkers

## Abstract

We aimed to assess some of the potential genetic pathways for cancer development from non-malignant intraductal papillary mucinous neoplasm (IPMN) by evaluating genetic mutations and methylation. In total, 46 dissected regions in 33 IPMN cases were analyzed and compared between malignant-potential and benign cases, or between malignant-potential and benign tissue dissected regions including low-grade IPMN dissected regions accompanied by malignant-potential regions. Several gene mutations, gene methylations, and proteins were assessed by pyrosequencing and immunohistochemical analysis. *RASSF1A* methylation was more frequent in malignant-potential dissected regions (*p* = 0.0329). *LINE-1* methylation was inversely correlated with *GNAS* mutation (*r* =  − 0.3739, *p* = 0.0105). In cases with malignant-potential dissected regions, *GNAS* mutation was associated with less frequent perivascular invasion (*p* = 0.0128), perineural invasion (*p* = 0.0377), and lymph node metastasis (*p* = 0.0377) but significantly longer overall survival, compared to malignant-potential cases without *GNAS* mutation (*p* = 0.0419). The presence of concordant *KRAS* and *GNAS* mutations in the malignant-potential and benign dissected regions were more frequent among branch-duct IPMN cases than among the other types (*p* = 0.0319). Methylation of *RASSF1A*, *CDKN2A*, and *LINE-1* and *GNAS* mutation may be relevant to cancer development, IPMN subtypes, and cancer prognosis.

## Introduction

An intraductal papillary mucinous neoplasm (IPMN) in the pancreas is a cystic tumor with unique histopathologic features, including massive dilatation of the pancreatic duct, mucin hypersecretion, and papillary epithelial projections into the pancreatic duct tributaries^1–3^. Some IPMNs progress to IPMN with associated invasive carcinoma (IC-IPMN), which is associated with a poor prognosis^4^. Pre-operative diagnosis of high-risk IPMNs is still challenging, although the International Consensus Guidelines for the Management of pancreatic IPMNs were revised in 2017^5^. The guideline defines main-duct (MD) IPMN patients and branch-duct (BD) IPMN patients based on worrisome features and high-risk stigmata to determine whether surgery is indicated. Although these criteria are useful for identifying patients recommended for surgery, their diagnostic accuracy for invasive IPMN before surgery needs to be improved^6^.

Characterization of the methylation patterns of genes implicated in human tumorigenesis may grant insight into the biology of pancreatic IPMNs^7^. *KRAS* and *BRAF* are two key oncogenes in the RAS/RAF/MEK/MAP-kinase signaling pathway and are also common gene mutations in colorectal cancer^8^. Pancreatic tumors reportedly harbor several gene aberrations, including those in *KRAS*, *GNAS*, and *BRAF*^9–11^. The early acquisition of a *KRAS* mutation is likely essential for triggering the adenoma-carcinoma sequence in pancreatic tumors^12^, and molecular profiling of *KRAS* and *GNAS* can help with determining whether invasive cancer in a pancreas with an IPMN is associated or concomitant^13^. Additionally, methylation of cyclin-dependent kinase inhibitor 2A (*CDKN2A*), a tumor suppressor gene that encodes P16 (or P16INK4a) and P14arf^14^, long interspersed nuclear element-1 (*LINE-1*) retrotransposition, a major hallmark of cancer accompanied by global chromosomal instability, genomic instability, and genetic heterogeneity^15^, and Ras association domain family member 1A (*RASSF1A*), a tumor-suppressor gene frequently inactivated in various human cancers^16,17^ has been studied in pancreatic tumors^14,18,19^. However, few studies have examined their methylation status in IPMN cases. Moreover, some IPMNs express P16 and P53^9,20^, which are encoded by *CDKN2A* and *TP53*, respectively. These gene and protein features may be linked to the clinical course of an IPMN, providing insight into its progression and enabling prediction of malignant transformation.

We assessed some of the potential genetic pathways for cancer development from non-malignant IPMN and evaluated the clinicopathological characteristics of IPMNs with based on genetic mutation and methylation profiling using pyrosequencing and immunohistochemical analysis.

## Methods

### Case selection

In total, 13 cases of IPMN with associated invasive carcinoma (IC-IPMN), 5 cases of IPMN with high-grade dysplasia (HG-IPMN, also known as carcinoma in situ), and 15 cases of sporadic IPMN with low-grade dysplasia (LG-IPMN) were retrieved from the pathology files of the Department of Experimental Pathology and Tumor Biology, Nagoya City University Graduate School of Medical Sciences. All tumor samples comprised resected, formalin-fixed, paraffin-embedded (FFPE) tissues. Informed consent was obtained, and the study was approved by the Institutional Review Board of Nagoya City University (approval no. 60-00-0990) and conducted in accordance with the Declaration of Helsinki. Clinicopathologic data were obtained from medical records. All hematoxylin and eosin (H&E)-stained slides were reviewed by three authors (K.M., G.A., and H.K.) blinded to the clinical information.

**Figure 1:**
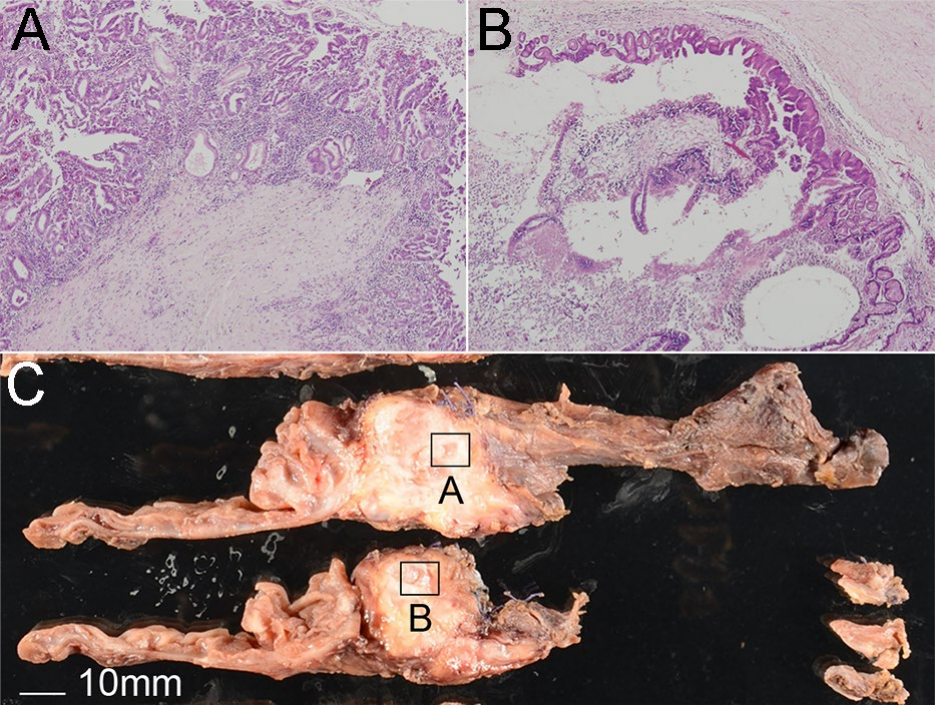
Representative images of the positional relationships between malignant-potential IPMN ((**A**), HG-IPMN in the image) and A-IPMN (**B**) dissected regions. Original magnification, × 40. Scale bar = 10 mm. A-IPMN was defined as a benign IPMN dissected region, as close as possible to the malignant-potential IPMN (within one or two slices).

Other LG-IPMN spots were chosen from the low-grade IPMN lesion involved in the original malignant-potential IPMN, as close as possible to, and ultimately one or two slides away from, the original 18 IC-IPMN and HG-IPMN lesions. They were dissected and collected as accompanying LG-IPMN (A-IPMN) samples. Representative images of the positional relationship between the malignant-potential and A-IPMN dissected regions are shown in Fig. 1. According to radiographic images and pathological findings, all IC-IPMNs were diagnosed with IPMN-derived carcinoma, which is different from concomitant invasive carcinoma^21^.

### Clinicopathologic data

The following clinicopathologic factors were analyzed: age, sex, primary tumor site (head, body/tail, or multifocal), tumor type (MD-IPMN, BD-IPMN, or mixed), tumor size, main pancreatic duct (MPD) dilatation, IPMN subtype, overall survival (OS), presence of mural nodules, and lymph node metastasis, vascular invasion, and perineural invasion status.

### DNA extraction and bisulfite treatment

All cases were manually macrodissected (approximately 10 × 10 mm) from tissues under a microscope (Eclipse 80i, Nikon, Tokyo, Japan) using a fine needle, and DNA was isolated from FFPE sections using the QIAamp DNA FFPE Tissue Kit (Qiagen, Hilden, Germany) and Maxwell 16 FFPE Tissue LEV DNA Purification Kit (Promega Corporation, Fitchburg, WI). A NanoDrop™ ND-1000 spectrophotometer (Thermo Fisher Scientific, Waltham, MA) was used to quantify the purified DNA. Bisulfite treatment was carried out as described previously^22^.

### DNA methylation analysis

DNA methylation was analyzed using bisulfite pyrosequencing as described previously^23,24^. Briefly, genomic DNA (1 μg) was modified with sodium bisulfite using an EpiTect Bisulfite kit (Qiagen). Pyrosequencing was carried out using a PSQ 96MA system (Qiagen) with a Pyro Gold Reagent kit (Qiagen), and the results were analyzed using Pyro Q-CpG software (Qiagen). Methylation of *CDKN2A*, *LINE-1*, and *RASSF1A* was analyzed using bisulfite pyrosequencing. Primer sequences are shown in Supplementary Table S1. A cut-off value of 10% was used to determine whether the *CDKN2A* and *RASSF1A* genes were methylation-positive as described previously^25–27^. *LINE-1* methylation was analyzed quantitatively.

### Analysis of *KRAS*, *BRAF*, and *GNAS* mutations

Mutations in *KRAS* (codons 12 and 13 of exon 2), *BRAF* (V600E), and *GNAS* (codon 201 of exon 8) were examined using a PyroMark Q24 pyrosequencer as described previously^28,29^. Each reaction contained 1× PCR buffer, 1.5 mM MgCl_2_, 0.2 mM each dNTP, 5 pmol forward primer, 5 pmol reverse primer (biotinylated), 0.8 U HotStarTaq DNA polymerase (Qiagen), 10 ng of template DNA, and dH_2_O to a final volume of 25 μL. Cycling conditions were as follows: 95 °C for 15 min; 38 cycles of 95 °C for 20 s, 53 °C for 30 s, and 72 °C for 20 s; and a final extension at 72 °C for 5 min, followed by holding at 8 °C. Following amplification, 10 μL of biotinylated PCR product was immobilized on streptavidin-coated Sepharose beads (streptavidin Sepharose high performance; GE Healthcare Bio-Sciences Corp., Piscataway, NJ) and washed in 70% EtOH. The purified biotinylated PCR products were loaded into the PyroMark Q24 system (Qiagen) with PyroMark Gold Reagent (Qiagen) containing 0.3 μM sequencing primer and annealing buffer. KRAS Pyro^®^ (Qiagen) and BRAF Pyro^®^ (Qiagen) were used to detect the *KRAS* and *BRAF* mutations, respectively, and the *GNAS* primer sequences are shown in Supplementary Table S1. A cut-off value of 10% was used to determine whether the genes were mutation-positive as described previously^30^.

### Immunohistochemistry

Tissue sections were deparaffinized and rehydrated. After antigen retrieval using heat treatment, immunohistochemistry (IHC) was performed using an automated immunostainer (Bond-Max, Leica Biosystems, Wetzlar, Germany) and monoclonal antibodies against RASSF1 (clone EPR7127, Abcam, Cambridge, UK; 1:100), P16 (clone E6H4, Ventana, Tucson, AZ; 1:1), P53 (clone DO7 NCL-L-p53-DO7, Leica Biosystems; 1:800). The tissue was considered to express RASSF1 and P16 when the stain levels for these proteins were equal to those seen in a normal pancreatic duct and a homogenous staining P53 IHC pattern in the epithelium was considered to reflect the expression of P53. For all IHC staining, the expression of protein in > 10% of epithelium from the dissected epithelium was considered a positive result^31–34^.

### Statistical analysis

Statistical analyses were performed using non-parametric tests. Continuous data are given as median values with ranges or means with SDs. Statistical evaluation of data from two groups was performed using the χ^2^ test, Fisher exact test, or Mann–Whitney U test for unpaired cases. The OS was measured from the date of surgery or diagnosis to the date of death from any cause. Patients not known to have died were censored on the date of their last follow-up. Survival curves were plotted using the Kaplan–Meier method and compared using the log-rank test. A two-sided *p*-value < 0.05 were considered significant. Correlations of methylation levels with other biological features were evaluated using Spearman’s rank-order correlation. Statistical analysis was performed using Prism 8 software (GraphPad Software, San Diego, CA).

## Results

### Patient characteristics

This study included 13 IC-IPMN, 5 HG-IPMN, 15 LG-IPMN, and 13 A-IPMN cases. According to the World Health Organization classification scheme^35^ and a previous study^36^, IPMN cases were classified into malignant-potential IPMN (IC-IPMN and HG-IPMN) and LG-IPMN cases. The patients’ characteristics are summarized in Table 1. The average age of the malignant-potential IPMN and LG-IPMN cases was 69 (range 55–84) and 68 (43–80) years, respectively. The malignant-potential IPMN cases included 8 males and 10 females, and the LG-IPMN cases included 13 males and 2 females. There were eight MD-IPMN cases (44%) among the malignant-potential IPMN cases, and eight of the LG-IPMN cases were also MD-IPMN cases (53%). Pathological examination of resected IC-IPMN tissues detected perivascular invasion and perineural invasion in 7 (53%) and 6 (46%) cases, respectively. Regarding therapeutic approaches, of 13 IC-IPMN patients, 4 patients received chemotherapy, 1 received radiotherapy, and 1 patient received chemoradiotherapy after tumor resection. Among the malignant-potential IPMN cases, the follow-up period ranged from 3 to 118 months. The overall 1-year survival rate of malignant-potential IPMN patients was 77%, with a median survival duration of 47 months. No significant differences in the patients’ characteristics were evident between the malignant-potential and LG-IPMN cases, except the proportion of males (44% *vs.* 86%, *p* = 0.0272).

**Table 1:** Patient characteristics.

	Malignant-potential IPMN (n = 18)		LG-IPMN (n = 15)		*p*
	IC-IPMN (n = 13)	HG-IPMN (n = 5)			
Age (mean [range])	69 (55–84)	71 (68–76)	68 (43–80)		NS*
Sex (male/female)	6/7	2/3	13/2		0.0272*
**Tumor location, n (%)**					
Head	6 (46)	3 (60)	7 (46)		NS*
Body or tail	6 (46)	1 (20)	8 (53)		
Multifocal	1 (7)	1 (20)	0 (0)		
**IPMN type, n (%)**					NS*
MD-IPMN	6 (46)	2 (40)	8 (53)		
BD-IPMN	6 (46)	1 (20)	7 (46)		
Mixed	1 (7)	2 (40)	0 (0)		
**Tumor size (mm), n (%)**					NS*
< 30/ ≥ 30	6 (46)/7 (53)	3 (60)/2 (40)	9 (60)/6 (40)		
**Mural nodule, n (%)**					NS*
Enhanced /none or non-enhanced	0 (0)	1 (20)	3 (20)		
	13 (100)	4 (80)	12 (80)		
**MPD dilatation (mm), n (%)**					NS*
< 10/ ≥ 10	3 (23)/10 (76)	0 (0)/5 (100)	4 (26)/11 (73)		
**Stage, n (%)**					
IA/IB	3 (23)/2 (15)	–	–		
IIA/IIB	1 (7)/5 (38)	–	–		
III	2 (15)	–	–		
**Lymph node metastasis, n (%)**					
Yes/no	6 (46)/7 (53)	–			
**Perivascular invasion, n (%)**					
Yes/no	7 (53)/6 (46)	–	–		
**Perineural invasion, n (%)**					
Yes/no	6 (46)/7 (53)	–	–		

Histopathologic type	Malignant-potential IPMN dissected regions (n = 18)		Benign IPMN dissected regions (n = 28)		
	IC-IPMN (n = 13)	HG-IPMN (n = 5)	LG-IPMN (n = 15)	A-IPMN (n = 13)	
Gastric, n (%)	6 (33^†^)	0 (0^†^)	12 (42^‡^)	6 (21^‡^)	NS^§^
Intestinal, n (%)	5 (27^†^)	4 (22^†^)	3 (10^‡^)	7 (25^‡^)	NS^§^
Pancreatobilliary, n (%)	2 (11^†^)	1 (5^†^)	0 (0^‡^)	0 (0^‡^)	0.0255^§^

MD-IPMN main duct IPMN, BD-IPMN branch duct IPMN.

*p-value for comparisons of malignant-potential IPMN and LG-IPMN.

^†^Proportions among malignant-potential IPMN dissected regions.

^‡^Proportions among benign dissected regions.

^§^*p*-value for comparisons among malignant-potential IPMN (IC-IPMN and HG-IPMN) and benign IPMN (LG-IPMN and A-IPMN) dissected regions.

On the basis of histology, all dissected tissue regions were classified as malignant-potential (IC-IPMN or HG-IPMN) or benign (LG-IPMN or A-IPMN) and further subclassified into gastric (n = 24), intestinal (n = 19), or pancreatobiliary types (n = 3). All dissected regions of pancreatobiliary type were malignant-potential IPMN dissected regions and statistically more frequent compared with benign IPMN dissected regions (16% vs 0%, *p* = 0.0255).

### Pyrosequencing

**Figure 2:**
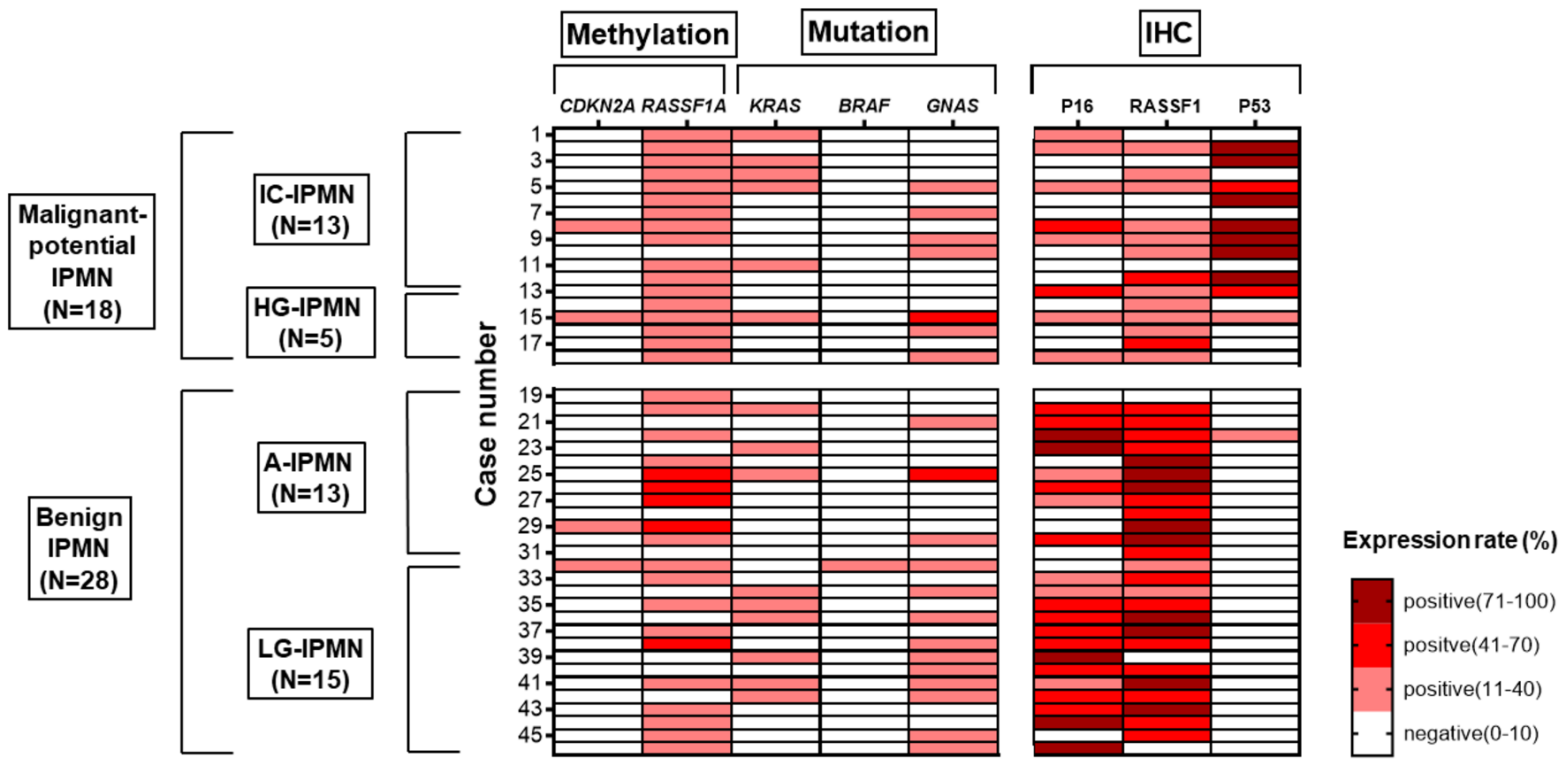
Heat map visualization of the results of pyrosequencing and IHC analyses. White cells, negative expression. Red or brown cells, positive expression. Because of the undetermined threshold, *LINE-1* methylation is not depicted.

Pyrosequencing analysis was performed in all cases (Supplementary Fig. S1), and the results are represented as heat maps (Fig. 2). The positive *RASSF1A* methylation rate differed significantly between the malignant-potential and benign IPMN dissected regions (94% *vs.* 67%, *p* = 0.0329). No significant difference in *CDKN2A* methylation (11% *vs.* 3%, *p* = 0.3121), *KRAS* mutation (33% *vs.* 35%, *p* = 0.8686), or *GNAS* mutation (38% *vs.* 53%, *p* = 0.3306) was evident between the two groups. *LINE-1* methylation levels have no significant difference between the two groups (*p* = 0.7173, Mann–Whitney U test). *BRAF* mutation was detected in one A-IPMN dissected region surrounding an HG-IPMN.

**Figure 3:**
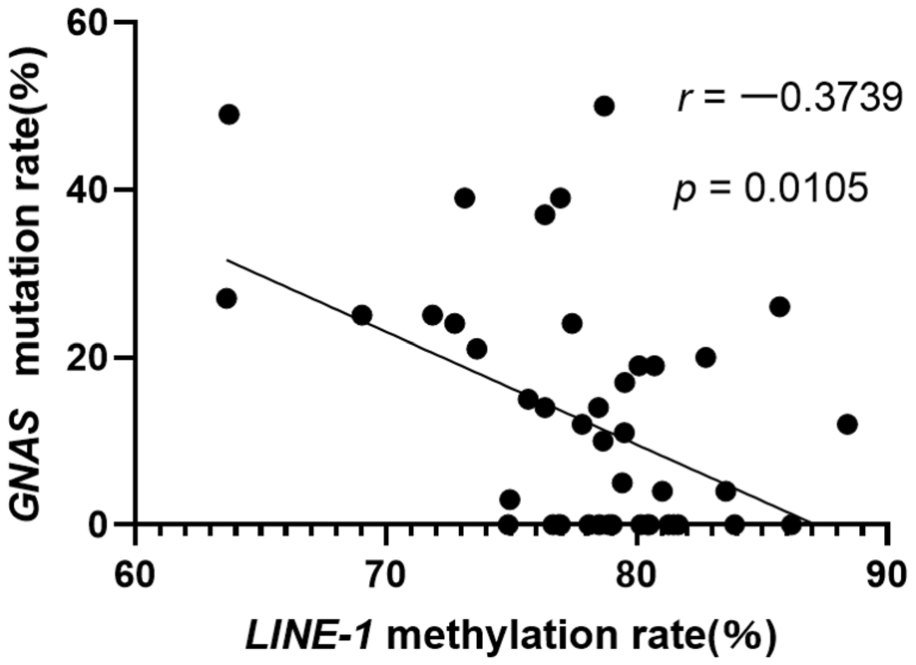
Scatter plots of the associations of the *LINE-1* methylation rate with the *GNAS* methylation rate.

*LINE-1* methylation was inversely correlated with *GNAS* mutation (*r* =  − 0.3739, *p* = 0.0105, Fig. 3), but it was not significantly correlated with either *KRAS* mutation (*r* =  − 0.1633, *p* = 0.2782) or *RASSF1A* methylation (*r* =  − 0.1151, *p* = 0.4463). Additionally, genetic aberrations in A-IPMN dissected regions showed no significance compared to LG-IPMN dissected regions.

### IHC analyses of P16, P53, and RASSF1

**Figure 4:**
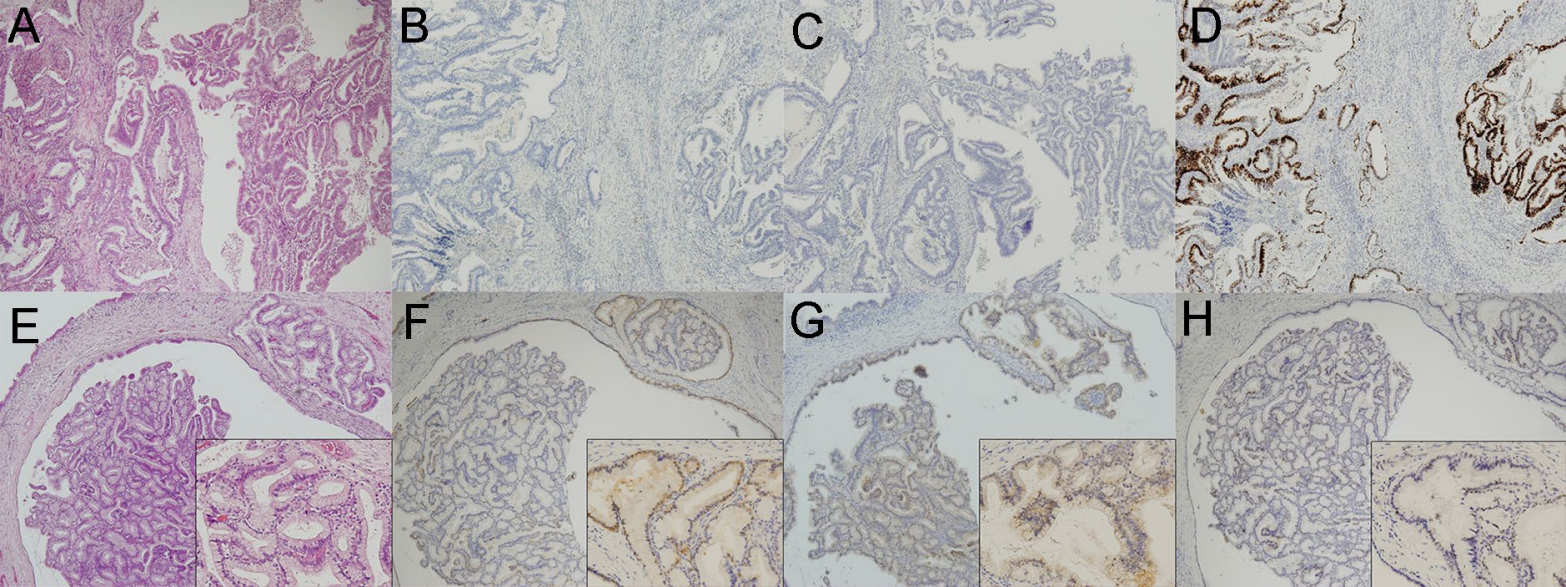
Representative images of a malignant-potential dissected region (**A–D**) and benign dissected region (**E–H**) showing H&E staining (**A,E**), and P16 (**B,F**), RASSF1 (**C,G**), and P53 (**D,H**) expression. Original magnification, × 40; inset magnification, × 200.

Representative images of the malignant-potential and benign IPMN dissected regions are shown in Fig. 4A–H. Typically, the malignant-potential IPMN dissected regions (Fig. 4A) were P16 negative (Fig. 4B), RASSF1 negative (Fig. 4C), and P53 positive (Fig. 4D), whereas the benign IPMN dissected regions (Fig. 4E) were P16 positive (Fig. 4F), RASSF1 positive (Fig. 4G), and P53 negative (Fig. 4H). The IHC results are shown in Fig. 2.

P16 positivity according to IHC was more frequent among benign than malignant-potential IPMN dissected regions (82% *vs.* 44%, *p* = 0.0078). The rate of *CDKN2A* methylation was inversely correlated with the rate of P16 IHC expression *(p* = 0.0024, *r* =  − 0.4375). Although there was no significant correlation between the rate of *RASSF1A* methylation and the rate of RASSF1 expression (*p* = 0.3588), 9 of 10 (90%) *RASSF1A* hypomethylation cases were positive for RASSF1 according to IHC. The rate of P53 positivity according to IHC, by contrast, was more frequent among malignant-potential IPMN dissected regions than benign IPMN dissected regions (55% *vs.* 3%, *p* < 0.0001).

The rates of P16, RASSF1, and P53 positivity according to IHC did not differ significantly across clinicopathological parameters or between LG-IPMN and A-IPMN dissected regions.

### Clinicopathologic associations of pyrosequencing and IHC outcomes

**Figure 5:**
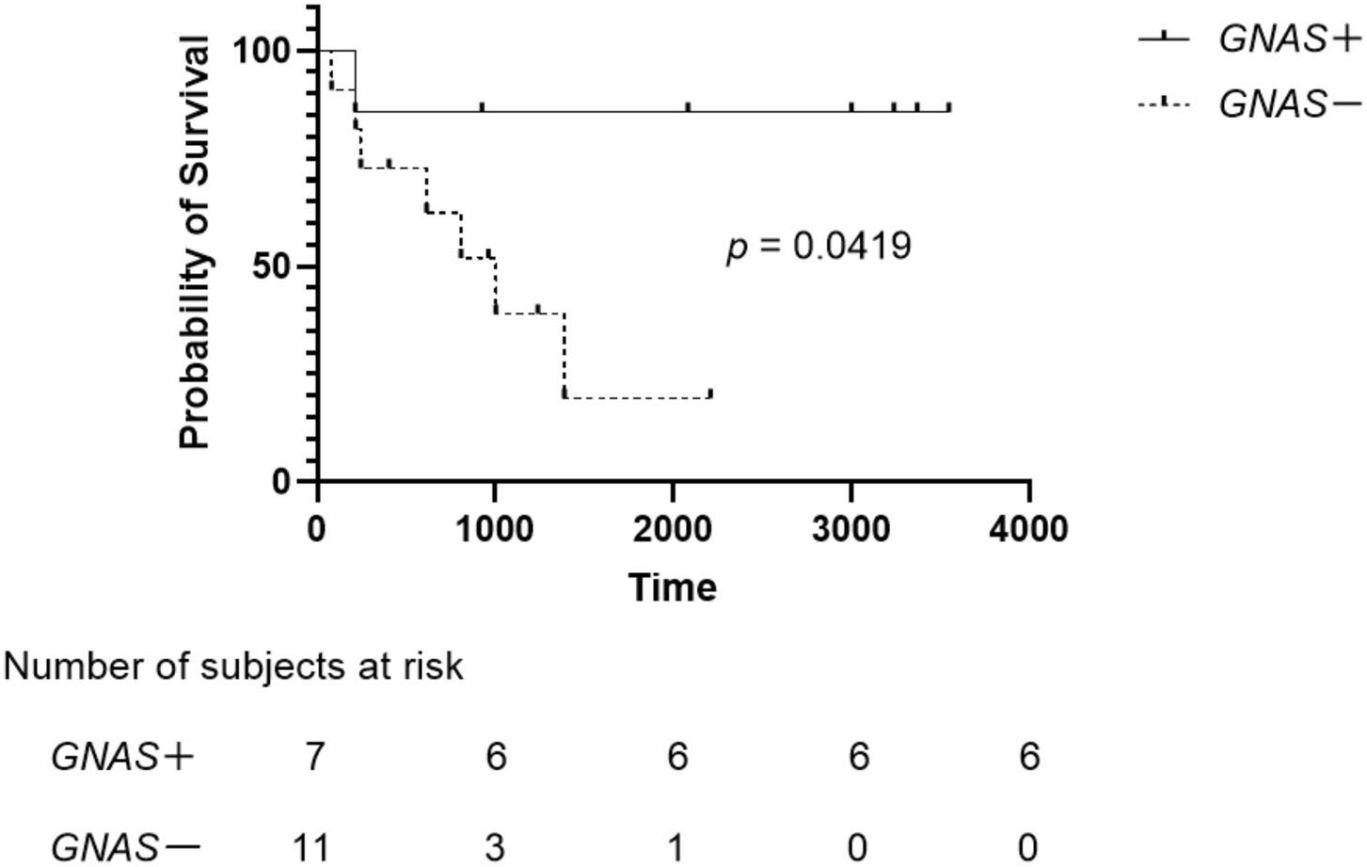
Overall survival of patients with (solid line) and without (dotted line) *GNAS* mutation in the malignant-potential IPMN dissected regions. Survival curves were plotted using the Kaplan–Meier method and were compared using the log-rank test.

The relationships between clinicopathological parameters, methylation and mutation status, and IHC results are shown in Table 2. Tumor size, the presence of mural nodules, and MPD diameter were compared among all cases; histological types of IPMN were compared among all tissue dissected regions; and the status of lymph node metastasis, perivascular invasion, and perineural invasion was compared among malignant-potential IPMN dissected regions. For all dissected regions, *CDKN2A* methylation was more frequent in intestinal-type dissected regions than in gastric-type dissected regions (15% *vs.* 0%; *p* = 0.0436). Among the malignant-potential IPMN dissected regions, *GNAS* mutation was less frequent among those with perivascular invasion compared to those without (0% *vs.* 63%, *p* = 0.0128), dissected regions with perineural invasion (0% *vs.* 58%; *p* = 0.0377), or dissected regions exhibiting lymph node metastasis (0% *vs.* 58%; *p* = 0.0377). Furthermore, 7 cases of malignant-potential dissected regions with a *GNAS* mutation had a significantly longer OS than 11 cases of malignant-potential dissected regions without a *GNAS* mutation (undefined days *vs.* 1004 days; *p* = 0.0419) (Fig. 5). The Histopathologic type of IPMN did not affect the prognosis of malignant-potential IPMN.

**Table 2:** Relationships between the clinicopathologic parameters and methylation, mutation, and IHC results.

Clinicopathologic features	n	*CDKN2A* methylation		*RASSF1A* methylation		*KRAS* mutation		*GNAS* mutation		P16 IHC		P53 IHC	
		n (%)	*p*	n (%)	*p*	n (%)	*p*	n (%)	*p*	n (%)	*p*	n (%)	*p*
**Clinical factors**													
Tumor size (mm)*													
< 30	19	0 (0)	NS	15 (78)	NS	9 (47)	NS	12 (63)	NS	12 (63)	NS	4 (21)	NS
≧ 30	14	2 (14)		12 (85)		5 (35)		6 (42)		10 (71)		6 (42)	
Mural nodule*													
Enhanced	11	0 (0)	NS	10 (90)	NS	4 (36)	NS	6 (54)	NS	7 (63)	NS	4 (36)	NS
None/non-enhanced	22	2 (9)		17 (77)		10 (45)		12 (54)		15 (68)		6 (27)	
MPD dilatation (mm)*													
< 10	7	0 (0)	NS	5 (71)	NS	4 (57)	NS	3 (42)	NS	6 (85)	NS	1 (14)	NS
≧ 10	26	2 (7)		22 (84)		10 (38)		15 (57)		16 (61)		9 (34)	
**Pathologic factors**													
Histologic types^†^													
Gastric	24	0 (0)	0.0436^§^	21 (87)	NS	9 (37)	NS	9 (37)	NS	19 (79)	NS	5 (20)	NS
Intestinal	19	3 (15)		12 (63)		4 (21)		12 (63)		10 (52)		5 (26)	
Pancreatobiliary	3	0 (0)		3 (100)		1 (33)		1 (33)		1 (33)		1 (33)	
Lymph node metastasis^‡^													
Yes	6	1 (16)	NS	6 (100)	NS	1 (16)	NS	0 (0)	0.0377	3 (50)	NS	5 (83)	NS
No	12	1 (8)		11 (91)		5 (41)		7 (58)		3 (25)		5 (41)	
Perivascular invasion^‡^													
Yes	7	1 (14)	NS	7 (100)	NS	3 (42)	NS	0 (0)	0.0128	4 (57)	NS	4 (57)	NS
No	11	1 (9)		10 (90)		3 (27)		7 (63)		4 (36)		6 (54)	
Perineural invasion^‡^													
Yes	6	0 (0)	NS	4 (66)	NS	2 (33)	NS	0 (0)	0.0377	3 (50)	NS	4 (66)	NS
No	12	2 (16)		9 (75)		4 (33)		7 (58)		5 (41)		6 (50)	

No significant differences were observed for *LINE-1* methylation, *BRAF* mutation, or the IHC results for RASSF1.

*All cases.

^†^All dissected tissue regions.

^‡^Only malignant-potential cases.

^§^*p*-value for comparison between gastric and intestinal types.

*RASSF1A* methylation, *LINE-1* methylation, *KRAS* mutation, and *BRAF* mutation status as well as the P16, RASSF1, and P53 positivity rates according to IHC did not differ significantly across clinicopathological parameters.

### Methylation and mutation differences in two dissected regions from the same case

To explore malignant initiation and transformation, the methylation and mutation status was compared between malignant-potential IPMN (IC-IPMN and HG-IPMN) and benign A-IPMN dissected regions obtained in pairs from 11 IC-IPMN and 2 HG-IPMN cases (Table 3). Overall, 5 out of 13 (38%) malignant-potential dissected regions harbored the same *KRAS* and *GNAS* mutations. Cases harboring concordant sequences of *KRAS* and *GNAS* mutations between malignant-potential IPMN and A-IPMN dissected regions were more frequent among BD-IPMN cases than among MD-IPMN and mixed IPMN cases (80% *vs.* 12%, *p* = 0.0319).

**Table 3:** Patterns of methylation and mutation results for malignant-potential IPMN and A-IPMN dissected regions accompanied by malignant-potential IPMN dissected regions.

Case #	Subtype		Malignant-potential IPMN dissected region					A-IPMN dissected region				
			*KRAS* Codon 12		*GNAS* V600E		Methylation positive	*KRAS* Codon 12		*GNAS* V600E		Methylation positive
			Sequence	%	Sequence	%		Sequence	%	Sequence	%	
**1**	BD-IPMN	**Invasive**	**GGT → GTT**	**16**	**WT**	**0**	***RASSF1A***	**GGT → GTT**	**17**	**WT**	**0**	***RASSF1A***
**2**		**Invasive**	**GGT → GTT**	**20**	**WT**	**0**	***RASSF1A***	**GGT → GTT**	**5**	**WT**	**0**	
**3**		**Invasive**	**GGT → GTT**	**3**	**WT**	**0**	***RASSF1A***	**GGT → GTT**	**8**	**WT**	**0**	***RASSF1A***
**4**		**Invasive**	**GGT → GTT**	**6**	**WT**	**0**	***RASSF1A***	**GGT → GTT**	**1**	**WT**	**0**	
5		Invasive	GGT → GAT	11	CGT → TGT	15	*RASSF1A*	GGT → AGT	4	CGT → TGT	4	*RASSF1A*
**6**	MD-IPMN	**Invasive**	**GGT → GAT**	**9**	**CGT → TGT**	**14**	***RASSF1A***	**GGT → GAT**	**37**	**CGT → TGT**	**49**	***RASSF1A***
7		Invasive	WT	0	WT	0	*CDKN2A* *RASSF1A*	GGC → GAC	3	CGT → TGT	25	
8		Invasive	GGT → GTT	9	WT	0	*RASSF1A*	GGT → GAT	7	WT	0	*RASSF1A*
9		Invasive	GGT → GAT	4	CGT → TGT	39		GGT → AGT	4	CGT → TGT	5	*RASSF1A*
10		Invasive	GGT → GAT	18	WT	0	*RASSF1A*	GGT → TGT	1	WT	0	*RASSF1A*
11	Mixed-IPMN	Invasive	GGT → AGT	6	WT	0	*RASSF1A*	GGT → TGT	1	WT	0	
12		HG-IPMN	GGT → GAT	4	CGT → TGT	25	*RASSF1A*	GGT → GAT	3	CGT → TGT	19	*RASSF1A*
13		HG-IPMN	WT	0	WT	0	*RASSF1A*	GGT → CGT	3	CGT → TGT	12	*CDKN2A* *RASSF1A*

Cases with bold letters have concordant *KRAS* and *GNAS* sequences between malignant-potential IPMN and A-IPMN dissected regions.

WT,  wild type.

*RASSF1A* hypermethylation was present in four IC-IPMN dissected regions—two in BD-IPMN cases, one in a MD-IPMN case, and one in a mixed IPMN case, in which no hypermethylation existed in comparable A-IPMN dissected regions. No malignant-potential dissected region had a *GNAS* sequence different from that in a comparable A-IPMN dissected region, and the *KRAS* sequence was identical. The *BRAF* mutation, *CDKN2A* methylation, and *LINE*-1 methylation status did not differ significantly between the two dissected regions.

## Discussion

IPMNs are frequently encountered in clinical practice and are associated with a risk of malignancy. Risk stratification based on radiological characteristics has been proposed^5^. Research has focused on molecular biomarkers relevant to malignant transformation and clinical characteristics, with a few used in clinical practice. We performed pyrosequencing and IHC analysis of 46 dissected regions (13 IC-IPMN, 5 HG- IPMN, and 28 LG-IPMN including 13 A-IPMN dissected regions) in 33 IPMN cases. IPMN tissue harbors various kinds of dysplasia. Therefore, it is common for pathological studies to choose more than two separate IPMN lesions separately in a given case and genetically analyze all of chosen spots^1,13^.

Gene mutations and methylation analyzed by pyrosequencing included those for *CDKN2A*, *RASSF1A*, *LINE1*, *KRAS*, *BRAF*, and *GNAS*, which have been investigated in IPMN or pancreatic ductal adenocarcinoma^11,17,37–40^. The reason why we used a cut-off value of 10% is because we macrodissected the tissue samples, which included non-tumor cells such as lymphocytes, fibroblasts, and acinar cells. We assume the mixture of a variety of cells would lower the cut-off values of the pyrosequencing compared with other studies and other studies examined *RASSF1A* methylation or *KRAS* and *GNAS* mutations used cut-off values of 10% as well^25,26,30^. In addition, protein levels of P16, P53, and RASSF1 were examined using IHC^28^.

Importantly, IPMNs with *RASSF1A* methylation were detected in 36 of 46 IPMN dissected regions (78%) and were more frequent in malignant-potential IPMN dissected regions than in benign IPMN dissected regions. *RASSF1* is a putative tumor suppressor gene that controls tumor growth by inhibiting the *RAS* pathway^41,42^ and *RASSF1A*, one of the seven transcript isoforms of *RASSF1*^43^, is frequently inactivated via methylation^44^. *RASSF1A* hypermethylation was detected in 64% of primary pancreas adenocarcinomas^45^, similar to our finding of *RASSF1A* hypermethylation in IPMN cases. There is reportedly an inverse correlation between *RASSF1A* silencing and *KRAS* activation^45^, although we did not obtain such a result. Our data implicate *RASSF1A* hypermethylation in the malignant transformation of benign IPMN epithelium. Interestingly, two cases of IC-IPMN dissected regions with *RASSF1A* hypermethylation did not exhibit *RASSF1A* hypermethylation in A-IPMN dissected regions, but all dissected regions harbored the same *KRAS* and *GNAS* mutations. Therefore, *RASSF1A* hypermethylation may play an important role in the transformation of benign IPMN epithelium. Dissected regions with *RASSF1A* hypermethylation failed to show an inverse correlation with RASSF1 expression, indicating that other factors—such as gene mutations and methylation of other *RASSF1* isoforms—modulate RASSF1 protein synthesis^43^.

*GNAS* mutation was positively correlated with the OS of patients with malignant-potential cases. In short, IPMN patients without *GNAS* mutation had a poor prognosis, consistent with a previous report on 149 IPMN cases among which *GNAS* mutation was associated with prolonged survival^46^. Furthermore, *GNAS* mutation was less frequent in the IC-IPMN dissected regions with perineural or perivascular invasion than in those without, indicating that IC-IPMN without *GNAS* mutation can be aggressive. Mutations in *GNAS* at codon 201 have been identified as a hallmark molecular alteration in IPMNs with a prevalence of 66%^39^ and *GNAS* mutation is frequent in IPMN-associated adenocarcinoma^47,48^. Some IPMN cases without *GNAS* mutation may progress aggressively, which can be associated with other genes.

Genetic and epigenetic alterations inactivating *CDKN2A* are encountered in many cancers, including pancreatic cancer^49^. In this study, *CDKN2A* methylation had neither a prognostic association nor a high frequency in the malignant-potential IPMN cases. However, methylation was significantly more frequent in intestinal-subtype dissected regions, although the histopathologic type of IPMN did not affect the prognosis of malignant-potential IPMN, as a previous study stated^46^. These findings on *CDKN2A* methylation have not been reported previously, and further studies to clarify the mechanism and association are needed.

We evaluated the links to global DNA methylation of *LINE-1*, hypomethylation of which is a common epigenetic alteration in tumor cells^50^. *LINE-1* did not exhibit significant hypomethylation in the IPMN cases and had no clinicopathological significance itself, as reported for pancreatic cancer^19^. Furthermore, the transpositional activity of *LINE-1* is typically silenced by DNA methylation and *LINE-1* hypomethylation causes genomic instability, leading to genome-wide mutations, insertions, or deletions^50^, consistent with the inverse correlation observed between *LINE-1* methylation and *GNAS* mutation. Therefore, *LINE-1* methylation may indirectly affect the malignant transformation of IPMN epithelium.

We performed a P53 IHC study instead of focusing on *TP53*, a tumor suppressor gene that prevents serious DNA damage and carcinogenesis^51^. P53 is mutated in around 50% of human cancers^52^. The majority of mutations occur within its central core sequence-specific DNA-binding domain with six hot spots in codons, resulting in the production of conformationally aberrant P53 proteins (mutant P53). *TP53* hot-spot mutations account for 30% of those reported^53^. The most common *TP53* mutations not only impair its tumor-suppressor function (loss of function) but also confer novel pro-oncogenic potential on *TP53* (gain of function), markedly enhancing tumor progression and drug resistance^54^. Additionally, P53 IHC positivity is reportedly relevant to the metastasis or prognosis of pancreatic ductal adenocarcinoma^49,50,55–57^ and is associated with the prognosis of pancreatic ductal cancer^58^. Our data suggest that P53 IHC positivity is associated with malignant transformation of IPMNs, consistent with a previous report^59^.

We also examined the genetic pathways in two dissected regions from the same case. The dissected material contained a variety of cell types such as lymphocytes, fibroblasts, acinar cells, besides the target IPMN epithelium. Additionally, the proportion of neoplastic content is different and low in samples^60^, even though their sample sizes are same. However, the study did not compare subtle difference of genetic or epigenetic aberrations between malignant-potential dissected regions, and just compare between malignant-potential and benign dissected regions. Therefore, low neoplastic content did not influence our result that *RASSF1A* methylation is frequent in malignant-potential IPMN. Based on clonal relations of driver mutations, Omori et al. classified IPMN development into three types: a sequential subtype featuring less diversity in incipient foci with frequent *GNAS* mutations; a branch-off subtype featuring identical *KRAS* mutations with different *GNAS* mutations; and a de novo subtype harboring driver mutations absent from concurrent IPMNs^13^. Patients with the branch-off subtype had longer disease-free survival compared to those with the other two subtypes^13^. Our study showed that the BD-IPMN developed via cloning in a sequential manner with concordant sequences of the *KRAS* and *GNAS* mutations. This is reasonable because IC-IPMN derived from a BD-IPMN progresses from an IPMN located in a small area of the branch duct independent from the MPD and other branch ducts. By contrast, MD-IPMN or mixed-type IPMN progresses in a large area, including the MPD. Although no other clinicopathological differences were detected according to *KRAS* or *GNAS* mutation status, further studies with additional samples might clarify meaningful associations based on the mutation sequences. Furthermore, although all malignant-potential IPMNs in this study initially seemed to be IPMN-derived histologically, 61% of the malignant-potential dissected regions had *KRAS* sequences different from those of the comparable A-IPMN dissected regions. Because the early acquisition of a *KRAS* mutation triggers the adenoma-carcinoma sequence^12^, some malignant-potential IPMNs with different *KRAS* mutations from the adjacent LG-IPMN may develop into concomitant pancreatic cancer independently of the original IPMN.

This study had several limitations. Macrodissection mixed the extracted DNA of various cells, except the tumor epithelium, resulting in a lower cut-off value for the pyrosequencing analysis. The small number of patients studied might have biased the analyses and prevented multivariate analysis. Moreover, small number of HG-IPMN precluded from showing any statistical significances to identify genetic or epigenetic aberrations in only the pre-malignant lesions. *TP53*, mutations of which are common in pancreatic cancer, had too many hot spots for pyrosequencing. Therefore, we evaluated P53 expression using IHC. Further studies are necessary to confirm our findings.

In conclusion, we studied several gene mutations and methylation events using pyrosequencing and IHC. Several of the gene aberrations detected may be relevant to cancer development, IPMN subtypes, and cancer prognosis. The findings provide insight into cancer development from an IPMN and will facilitate clinical surveillance and treatment-related decision-making.

## Supplementary Information

Supplementary Information.
