# Supplementary material for: Relevance of gene mutations and methylation to the growth of pancreatic intraductal papillary mucinous neoplasms based on pyrosequencing
Source: Sci Rep. 2022 Jan 10;12:419. doi: 10.1038/s41598-021-04335-z (PMC8748617; doi:10.1038/s41598-021-04335-z)
Supplement: Supplementary file 1 — Supplementary Information. [file 41598_2021_4335_MOESM1_ESM.pdf]

Title:

Relevance of Gene Mutations and Methylation to the Growth of Pancreatic Intraductal Papillary Mucinous Neoplasms Based on Pyrosequencing

Go Asano, MD[1], Katsuyuki Miyabe, MD, PhD[1,2,*], Hiroyuki Kato, MD, PhD[3], Michihiro Yoshida, MD, PhD[1], Takeshi Sawada, MD, PhD[1], Yasuyuki Okamoto, MD, PhD[1], Hidenori Sahashi, MD[1], Naoki Atsuta, MD[1], Kenta Kachi, MD[1], Akihisa Kato, MD, PhD[1], Naruomi Jinno, MD, PhD[1], Makoto Natsume, MD, PhD[1], Yasuki Hori, MD, PhD[1], Itaru Naitoh, MD, PhD[1], Kazuki Hayashi, MD, PhD[1], Yoichi Matsuo, MD, PhD[4], Satoru Takahashi, MD, PhD[3], Hiromu Suzuki, MD, PhD[5], Hiromi Kataoka, MD, PhD[1]

Affiliation:

(1) Department of Gastroenterology and Metabolism, Nagoya City University Graduate School of Medical Sciences, Nagoya 467-8601, Japan

(2) Department of Gastroenterology, Japanese Red Cross Aichi Medical Center Nagoya Daini Hospital, Nagoya 466-8650, Japan

(3) Department of Experimental Pathology and Tumor Biology, Nagoya City University Graduate School of Medical Sciences, Nagoya 467-8601, Japan

(4) Department of Gastroenterological Surgery, Nagoya City University Graduate School of Medical Sciences, Nagoya 467-8601, Japan

(5) Department of Molecular Biology, Sapporo Medical University School of Medicine, Sapporo 060-8556, Japan

E-mail:

Go Asano; goasano@med.nagoya-cu.ac.jp

Katsuyuki Miyabe; kmiyabe@med.nagoya-cu.ac.jp

Hiroyuki Kato; h.kato@med.nagoya-cu.ac.jp

Michihiro Yoshida; mityoshi@med.nagoya-cu.ac.jp

Takeshi Sawada; sawada1046@gmail.com

Yasuyuki Okamoto; yasuokmt@gmail.com

Hidenori Sahashi; hsahashi@med.nagoya-cu.ac.jp

Naoki Atsuta; the_fighting_la_laa_palloza@yahoo.co.jp

Kenta Kachi; kachi208@gmail.com

Akihisa Kato; akihisa@med.nagoya-cu.ac.jp

Naruomi Jinno; naru1211@med.nagoya-cu.ac.jp

Makoto Natsume; makoto04130510@gmail.com

Yasuki Hori; yhori@med.nagoya-cu.ac.jp

Itaru Naitoh; inaito@med.nagoya-cu.ac.jp

Kazuki Hayashi; khayashi@med.nagoya-cu.ac.jp

Yoichi Matsuo; matsuo@med.nagoya-cu.ac.jp

Satoru Takahashi; sattak@med.nagoya-cu.ac.jp

Hiromu Suzuki; hsuzuki@sapmed.ac.jp

Hiromi Kataoka; hkataoka@med.nagoya-cu.ac.jp

***Corresponding author:**

Katsuyuki Miyabe, MD, PhD

Department of Gastroenterology and Metabolism, Nagoya City University

Graduate School of Medical Sciences

1, Kawasumi, Mizuho-Cho, Mizuho-Ku, Nagoya, 467-8601, Japan

E-mail: kmiyabe@med.nagoya-cu.ac.jp

Phone: +81-52-853-8211; FAX: +81-52-852-0952

Supplementary Table S1. Primer sequences in this study

Gene	Primer/target	Forward	Reverse	Product size (bp)
CDKN2A	Pyroseq PCR	5’-AGAGGAGGGGTTGGTTGGTTATTAG-3’	5'-Bio-ATCAACCGAAAACTCCATACTACTCC-3'	140
	Sequencing primer	5'-GGGAGTAGTATGGAGT-3		
	Target	5’-ATYGYGGTTTTTTTAGAGGATTTGAGGG-3’		
RASSF1A	Pyroseq PCR	5'-TTGAAGTCGGGGTTCGTTTTGTGGT-3'	5'-Bio-ACTCAAACTCCCCCGACATAACCC-3'	103
	Sequencing primer	5'-GTTAGCGTTTAAAGTTAG-3'		
	Target	5’-YGAAGTAYG-3’		
LINE-1	Pyroseq PCR	5’-TTTTGAGTTAGGTGTGGGATATA-3’	5’-Bio-AAAATCAAAAAATTCCCTTTC-3’	146
	Sequencing primer	5’-GGGTGGGAGTGAT-3’		
	Target	5’-TYGATTTTTTAGGTGYGTTYG-3’		
GNAS	Pyroseq PCR	5'-GGTTGGCTTTGGTGAGATCCATT-3'	5'-ACTTTGTCCACCTGGAACTTGGT-3'	107
	Sequencing primer	5’-GACCTGCTTCGCTGC-3’		
	Target	5’-YRTGTCCTG-3’<br>5’-YGTGTCCTG-3’<br>5’-CRTGTCCTG3’		

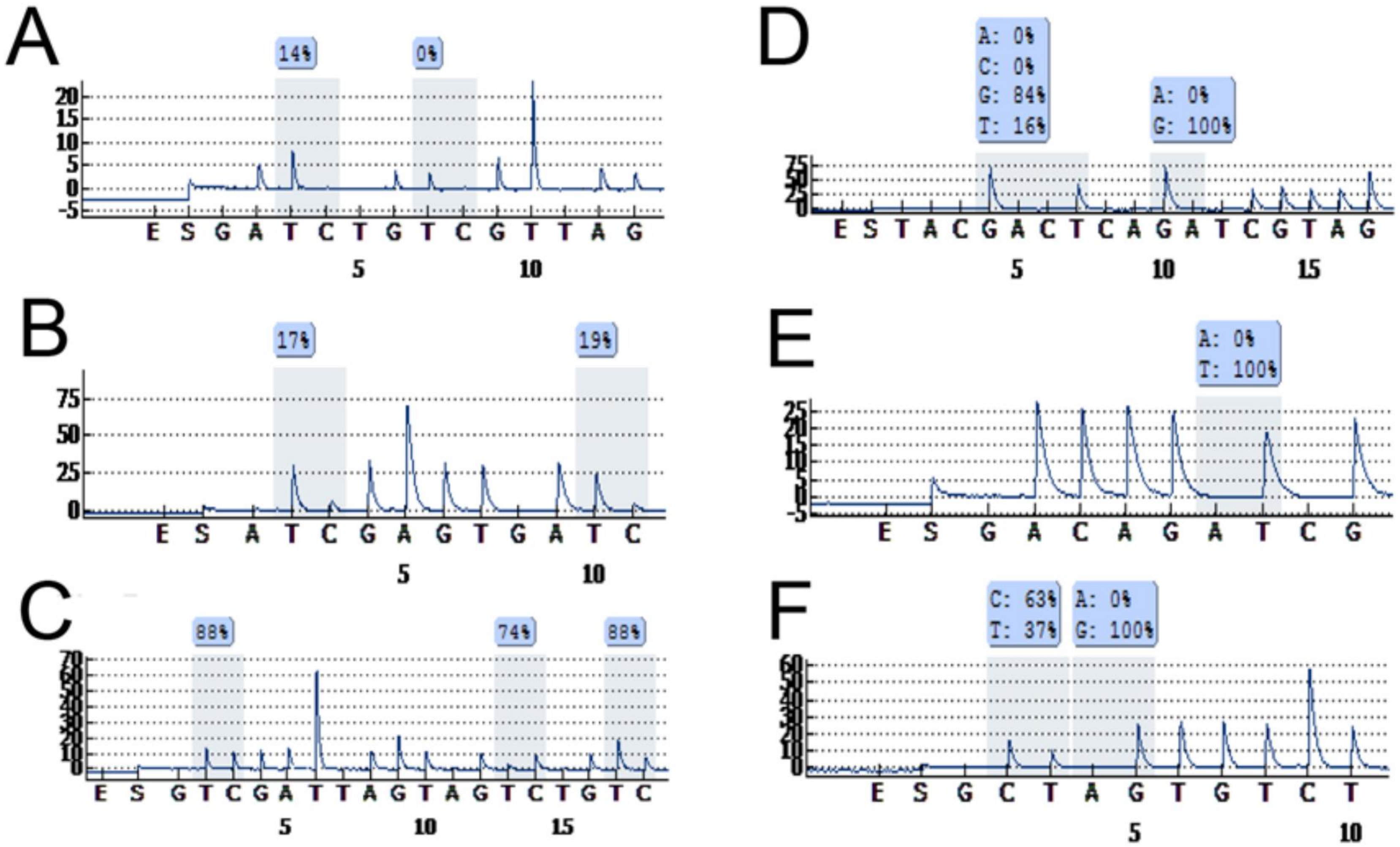

Supplementary Figure S1. Representative pyrosequencing results of *CDKN2A* methylation (A), *RASSF1A* methylation (B), and *LINE-1* methylation (C), *KRAS* mutation of codon 12 and codon 13 (D), *BRAF* mutation of V600E (E), and *GNAS* mutation of codon 201 (F). The percentage methylation at each CpG site is shown on the top (A,B,C). The allele frequencies are given above the indicated sites (D, E, F).
